# Spectrum of substance use precipitating rehabilitative services among adult patients in the university of Port Harcourt teaching hospital

**DOI:** 10.4314/ahs.v23i3.82

**Published:** 2023-09

**Authors:** Chukwuma Ugochukwu Okeafor, Irene Esu

**Affiliations:** 1 University of Port Harcourt, Department of Neuropsychiatry; 2 Federal Ministry of Health, Department of Public Health

**Keywords:** substance use, alcohol, cannabis, substance use, rehabilitative care

## Abstract

**Background:**

World Health Organization ranks substance abuse as one of the top twenty health risk factors. It poses a serious public health crisis with a significant burden for affected individuals, families and health systems. This study aimed to identify the spectrum and pattern of substance use among patients receiving rehabilitative services.

**Methods:**

The study was a cross-sectional design involving 190 patients receiving rehabilitative care for substance use in the University of Port Harcourt Teaching Hospital. The Alcohol, Smoking and Substance Involvement Screening Test (ASSIST), a validated and reliable tool was employed to obtain data on substance use. Statistical analyses were performed at P<0.05.

**Results:**

The male to female ratio was 4:1. A higher proportion of the patients were within 18-35years category (81.6%; n=155). The commonly used substances were alcohol (90.5%; n=172), cannabis/marijuana (43.7%; n=83), prescription opioids (28.9%; n=55). cigarette (25.8%; n=49), and street opioids (15.8%; n=30). The prevalence of poly substance use was 68.4%(n=130). Age(p=0.033) and sex (being male) (p=0.002) were predictors for number of substances used.

**Conclusion:**

Almost all the patients receiving drug rehabilitative service used alcohol, and approximately 7 in 10 used more than one substance. The need to inculcate harm reduction in the management protocol is therefore paramount.

## Introduction

Substance use including alcohol use is often initiated in adolescence and peaks during adulthood, resulting in an increased risk for health and other complications.^1^ It causes a wide range of undesirable effects, which often depends on the specific drug or drugs used, the frequency and intensity of drug use, the person's health and other relevant factors.^2^ The National Institute of Drug Abuse (NIDA) defines substance addiction as a chronic disorder of the brain associated with a compelling desire to seek and use alcohol and drugs in spite of the endangering effects to the body of the victim.^3^ Substance abuse including alcohol is referred to as a brain disease because these substances change the brain structure and how it works.^3^ According to the American Psychiatry Association (APA), people with alcohol and substance addiction have distorted thinking, behaviour and body functions. It makes the affected persons have an intense craving for the drugs and makes it hard to discontinue their use.^4^

Notably, the recurrent use of alcohol and/or drugs cause clinically and functionally significant impairment, resulting in a myriad of longstanding health problems, disability, and a failure to meet major responsibilities at work, school, or home.^5^ Thus, they pose a serious public health crisis with a significant burden for individuals affected and their families.^6^ Caring for individuals with alcohol and substance addiction places a substantial burden on public health systems globally.^7^ This is due to the sad reality that few persons (only one out of six people worldwide and one out of 18 people in Africa) in need of drug rehabilitative care have access to treatment programmes.^8^

The United Nations Office on Drug and Crimes (UNODC) recommends the implementation of comprehensive, integrated and balanced national drug control strategies, policies and programmes in addressing and countering emerging and persistent challenges and threats of all aspects of world drug problems.^9^ The World Health Organization (WHO) has listed substance abuse as one of the top 20 health risk factors and adopted a public health approach to screening for substance use.^10^ Consequently, the Alcohol, Smoking and Substance involvement screening test (ASSIST) was developed by the WHO as a tool for substance and alcohol use screening among individuals.^11^

Identifying the pattern and spectrum of substances being used among persons seeking rehabilitative service is essential as this would not only expose the burden of the problem but also inform local, regional and national policy makers on type of measures to adopt. Notably, rehabilitative care services are commonly tailored to the type of substances being used, therefore a study assessing the spectrum of substances being used will aid planning and forecasting of required resources for rehabilitative services especially in economically disadvantaged settings. Also, harm Thus, this study sought to describe the spectrum and pattern of substance use among patients receiving rehabilitative care in a drug unit.

## Subjects and methods

### Study centre

The study was conducted in the University of Port Harcourt Teaching Hospital (UPTH). It is a tertiary hospital with 17 clinical department and 650 beds, offering healthcare services to Rivers State and surrounding states in the Niger-Delta region of Nigeria.

### Study design

A hospital based cross-sectional design was adopted.

### Ethical consideration

Ethical clearance certificate with number NHREC/05/01/2008B-FWA0002458-IRB00002323 was obtained from the Research and Ethics Committee of the University of Nigeria Teaching Hospital (UNTH) prior to the commencement of the study. The principles of ethics were upheld in the study.

### Study population

Adult patients undergoing drug rehabilitative service in the mental health department of UPTH, with no severe psychological disturbance precluding them from participation in the study, formed the study population for this research.

### Sample size calculation and sampling technique

The formula for cross-sectional design^12^ was used in the sample size calculation to obtain a value of 190, based on a standard normal deviate of 1.96, 14.4% prevalence of substance use among Nigerians according to UNODC,^13^ and tolerable error limit of 0.05. Systematic sampling was employed to select the patients receiving rehabilitative service to limit selection bias. The department has a daily average of 4-5 patients assessing rehabilitative services for alcohol and substance use. Thus, for the study, systematic sampling was employed based on a sampling interval of two, consequently every 2^nd^ patient was sampled daily in the study for the five-month data collection period.

### Study instrument

The Alcohol, Smoking and Substance Involvement Screening Test (ASSIST) version 3 was used in this research to obtain information on alcohol and substance use. The ASSIST version 3 obtains information on the following substances: tobacco, alcohol, cannabis, cocaine, amphetamine, inhalants, sedatives, hallucinogen, opioids, and other substance.^11^ The modified version of ASSIST further expands opioids into two groups namely: prescription opioids and street opioids.^14^ Thus, in the index study street opioids and prescription opioids were identified and presented separately. The ASSIST scoring provides information on levels of risk and the required intervention based on the risk level for the type of substance used.^11^ Risk is categorized as lower, moderate and high, and interventions required for each of the risk are no intervention, brief intervention and referral to specialist care respectively.1^1^,^15^ The tool is reliable, with an internal consistency (Cronbach's alpha) of more than 0.80 for the plurality of domains and a strong concurrent validity.^16^,^17^ ASSIST also demonstrates a good discriminative and predictive validity.^18^,^17^,^19^ For the index study, ASSIST was used to identify the spectrum of substances used by the respondents in the past three months. The risk was calculated based on participant's response to substance use generally and not for each specific substance.

### Data collection

A secure electronic data method was employed in data collection. The Kobo Collect software was used for data collection and transmission. The demographic questions and ASSIST questions were designed on the Kobo Collect software. It was interviewer-administered and took an average of 10minutes to complete the tool for each sampled participant.

### Statistical analysis

The Statistical Package for Social Sciences (SPSS) version 21 was used for statistical analysis. Pearson's Chi Square and Fisher's exact tests were used to determine significant differences in proportions. Fisher's exact test was employed when the expected cell value was below five in at least twenty percent of the crosstab cells. Data were tested for normality using Kolmogorov-Smirnov Statistics. Consequently, independent t-test and one-way Analysis of Variance (ANOVA) were employed in the comparison of mean number of substances used by demographic characteristics to determine significant differences. Pearson's correlation and simple linear regression analysis were employed to determine relationship between number of substances used and age. Statistical significance was set at alpha of 0.05. Statistically significant variables were entered into a multiple linear regression model to control for confounders and identify predictors. The dependent variable was number of substances being used, while the demographic findings comprised the independent variables. The regression coefficient (B) was used to explain the relationship between the dependent and independent variables. Confidence intervals were determined at the 95% level.

## Results

### Age and sex characteristics

This hospital-based study comprised 190 adults with mean age of 31.17±7.13years. The male to female ratio was 4:1. More than 80% of them were males and within the age bracket of 18-35 years. (Table 1) There was no significant difference between proportions by age categories and sex (*Chi Square*=1.396; P=0.237) as shown in table 1.

**Table 1:** Comparison of age categories between male and female patients receiving rehabilitative care for substance use in UPTH

Age category	Female n (%)	Male n (%)	Total n (%)
18 – 35 years	31 (88.6)	124 (80.0)	155 (81.6)
36 – 55 years	4 (11.4)	31 (20.0)	35 (18.4)

**Total**	**35 (100.0)**	**155 (100.0)**	**190 (100.0)**

*Chi Square=1.396; p=0.237*

### Spectrum of Substance use

Alcohol was the most commonly used substance 172(90.5%). Other commonly used substances were cannabis/marijuana 83(43.7%), prescription opioids 55(28.9%) and cigarette 49(25.8%). The least used substance was hallucinogens 7(3.7%). Six of the patients reported illicit intra-venous drug use (3.2%) (Table 2)

**Table 2:** Type of substances being used by patients receiving rehabilitative care, UPTH

Substances used*	Frequency (N=190)	Percent (%)
Alcohol	172	90.5
Cannabis /Marijuana	83	43.7
Prescription opioids	55	28.9
Cigarette	49	25.8
Street opioids	30	15.8
Tranquillizers / Sedatives	21	11.1
Cocaine	19	10.0
Prescription stimulants	17	8.9
Methamphetamine	14	7.4
Hallucinogens	7	3.7
Illicit Intra-venous drug use	6	3.2

* *Multiple responses apply*

### Substance use risk levels

**Figure 1:**
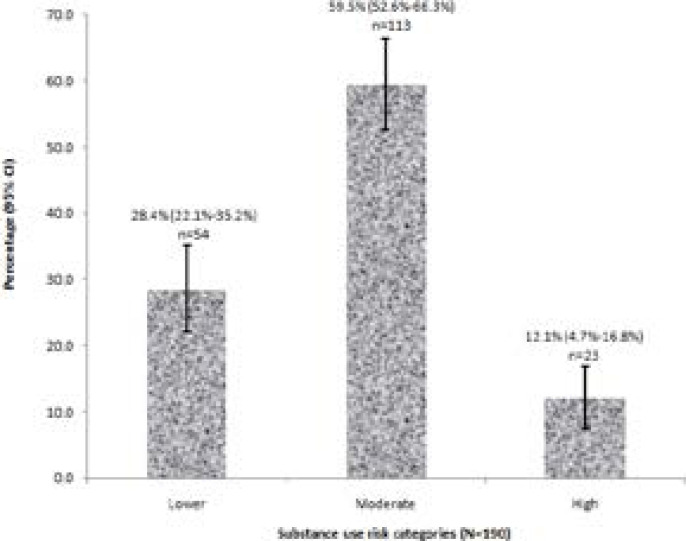
Substance use risk levels

Approximately 6 in 10 patients in the study had moderate risk levels (59.5%; 95% CI: 52.6%-66.3%; n=113). About one-quarter of the patients had ‘lower’ risk level, revealing that the majority three-quarters had moderate/high risk levels. The distribution of patients with lower, moderate and high-risk levels are presented in Figure 1.

### Comparison of number of substances being used by age and sex of patients

Table 3 shows the number of substances being used by male, female and overall patients in the study. Overall, the use of one substance had the highest proportion of 31.6% (n=60), while the least proportion of the patients used more than five substances (5.8%; n=11).

**Table 3:** Number of substance(s) being used by male and female patients receiving rehabilitative care for substance use in UPTH

Number of substance(s)	Female n (%)	Male n (%)	Total n (%)
One	16 (45.7)	44 (28.4)	60 (31.6)
Two	13 (37.1)	42 (27.1)	55 (28.9)
Three	3 (8.6)	21 (27.1)	24 (12.7)
Four	3 (8.6)	24 (15.5)	27 (14.2)
Five	0 (0.0)	13 (8.4)	13 (6.8)
More than five	0 (0.0)	11 (7.1)	11 (5.8)

**Total**	**35 (100.0)**	**155 (100.0)**	**190 (100.0)**

Fisher's exact=9.861; p=0.011* *Statistically significant

A total of 130 of the patients had poly substance use i.e., use of more than one substance (68.4%). In comparison to the females, higher proportion of males abused three substances (13.5% versus 8.6%), four substances (15.5% versus 8.6%), and five substances (8.4% versus 0.0%). The differences in the proportions between males and females was statistically significant (P=0.011).

**Figure 2:**
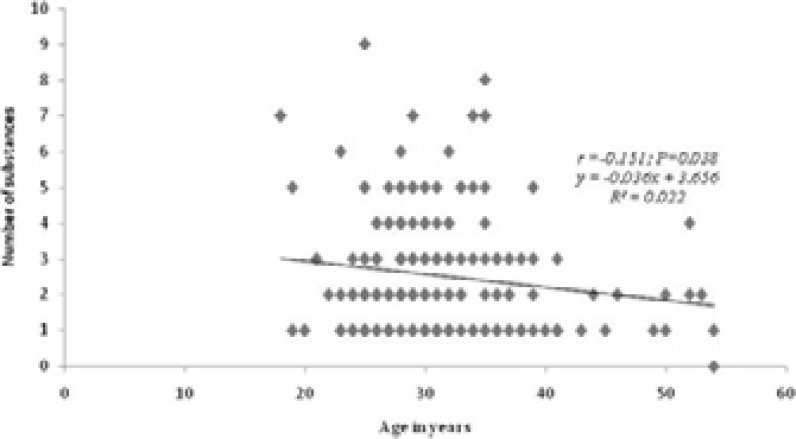
Correlation between number of substance(s) used and age of patients

There was a significant negative correlation between age and number of substances being used (r=-0.151; P=0.038) as shown in Figure 2. The simple linear regression analysis showed that for every unit increase in age, number of substances being used decreases by 0.036 (Figure 2)

### Other demographic characteristics of patients and number of substances being used

More than three-quarters (79.5%) were currently unmarried, and majority (90.6%) had secondary level and higher level of education. Concerning employment and economic status, highest proportion of the respondents were employed (70.5%), and above poverty line based on >$1/day (77.8%). (Table 4)

**Table 4:** Demographic characteristics and number of substances used by patients receiving drug rehabilitative service, UPTH

Variables	Frequency (%) (N=190)	Mean (SD)
**Age category**		
18 – 35 years	155 (81.6)	2.70 (1.69)
36 – 55 years	35 (18.4)	1.80 (1.05)
		t=3.010; p=0.003*
**Sex**		
Female	35 (18.4)	1.85 (1.03)
Male	155 (81.6)	2.68 (1.70)
		t=2.765; p=0.006*
**Marital Status**		
Currently married	39 (20.5)	1.92 (1.24)
Currently unmarried	151 (79.5)	2.69 (1.68)
		t=2.665; p=0.008*
**Educational level**		
Below secondary school level	18 (9.5)	2.72 (1.96)
Secondary level	78 (41.1)	2.54 (1.66)
Tertiary level	94 (49.5)	2.49 (1.54)
		*ANOVA=0.155; p=0.857*
**Employment status**		
Employed	134 (70.5)	2.36 (1.56)
Unemployed/student	56 (29.5)	2.95 (1.71)
		t=2.300; p=0.023*
**Income (NGN)**		
Below poverty line ((≤$1/day)	42 (22.1)	2.21 (1.66)
Above poverty line (>$1/day)	148 (77.8)	2.62 (1.61)
		*t=1.437; p=0.152*

* *Statistically significant*

The comparison of mean number of substances by demographic findings of the patients showed significantly higher number of substances being used by patients aged 18-35years (P=0.003), males (P=0.006), currently unmarried (P=0.008) and among students/unemployed (P=0.023) as shown in Table 4.

### Multivariate analysis of number of substances being used (dependent variable) and demographic findings (independent variables)

Age and sex remained significant predictors for substance use after adjusting for demographic characteristics of marital status and education. (Table 5) For every unit increase in age, the number of substances being used decreases by approximately 1unit (B=-0.791, 95%CI: -1.561, -0.067) as demonstrated in Table 5. Also, for sex, being male significantly increases number of substances used by approximately 1 unit (B=0.931, 95%CI: 0.329, 1.496).

**Table 5:** Multiple linear regression analysis model showing demographic factors related to the number of substances used by patients receiving drug rehabilitative service, UPTH

Variables	Number of substances being used			

	*β*	95% Confidence Interval for *β*		*p*

		Lower limit	Upper limit	
Age	-0.791	-1.516	-0.067	0.033*
Sex	0.913	0.329	1.496	0.002*
Marital status	-0.295	-0.988	0.399	0.403
Educational level	-0.152	-0.495	0.190	0.381

* *Statistically significant*

## Discussion

This study on the pattern and spectrum of substance use among patients receiving drug rehabilitative services showed that the highest proportion of them were within the 18-35years age group, males, unmarried, above secondary level of education, employed and had income above poverty line(>$1/day). The preponderance of young age category and males in substance abuse in this study is in keeping with several studies,^20^,^21^,^22^ and thus highlights the need for targeted drug abuse prevention models tailored at this population. The male predominance among patients receiving drug rehabilitative service may be described as unsurprising; however, the finding of approximately one-fifth of substance abusers being females signals a call for greater efforts in curbing this seemingly rising trend. Notably, a higher proportion of substance abuse has been reported among female population in a Zambian study,^23^ although in contrast with the index study, it however reiterates the need to institute drug abuse preventive mechanisms among female population. Apparently, the finding of greater proportion of substance users in the group that were more educated, employed and have better income in this study could be described as an iceberg phenomenon, as their demographic predisposition enables them to seek health care service as they can afford rehabilitative care bills. It is sad to note that in Nigeria and most parts of sub-Saharan Africa, health care expenditure is mainly via out-of-pocket mechanisms, therefore only persons who can afford the hospital bills seek such services.^24^,^25^ So, substance users who have low educational level and poorer socio-economic class may fail to present to the hospital for rehabilitative service. Hence, to obtain unbiased socio-demographic characteristics of substance users, a community-based study and not an institutional-based one such as the index study could be adopted.

Concerning commonly used substances, alcohol and cannabis/marijuana had highest frequencies, which is in tandem with a study in south-western Nigeria 26 and a national survey of substance use carried out in Nigeria.^21^ The finding in index study, showing that nine in 10 patients receiving drug rehabilitative service abused alcohol, possibly reflects the wide spread alcohol brand advertisements on both mass and social media. These adverts do not take into cognisance the negative psychological influence of alcohol on the user. Mental health experts should utilize mass media in enlightening the society of the ills of drug abuse especially among the youths. The finding of prescription opioids being the third commonly abused substance in this study is in contrast with another study in Saudi Arabia that found it among the least frequent used substance.^27^ Nonetheless, other studies in Nigeria and Africa have reported it as a commonly abused substance.^20^,^21^,^23^ Although, the index study did not investigate the circumstances regarding prescription opioids abuse, the findings expose the need for physicians and pharmacists, who are saddled with the responsibility of prescribing and dispensing opioids prescription respectively to remain vigilant. Also, regulations targeted at limiting access to prescription opioids are necessary to curb abuse. The dichotomization of opioids into prescription and street opioids in this study made it the third and fifth commonly used substance respectively. However, eliminating this dichotomy, makes opioids the most prevalent substance used after alcohol. This revelation uncovers the need for public health advocacy campaigns as well as the expansion of rehabilitative services to address this burden in the nation.

Although, being categorized as lower risk by ASSIST scoring implies no need for an intervention,^11^ our study however highlights the need for further evaluation among patients identified as lower risk. This is necessitated by the finding that about one-quarter of the patients who were categorized as lower risk still required rehabilitative care services in the study centre. Noteworthy, application of ASSIST tool even in non-health settings such as educational institutions could help to rapidly identify substance users that will need intervention. The finding of moderate and high-risk levels in about three-quarter of the patients further highlights the relevance of harm reduction in rehabilitative service. The harm reduction model is a public health approach that seeks to manage risk behaviours by implementing several strategies and actions to reduce the negative outcomes of such behaviours.28 Notably, harm reduction is a vital part of the care protocol in the mental health department of the study centre. Although the study did not identify substance abuse and drug dependence, but rather substance use among patients receiving rehabilitative services, it highlights the need for consideration of harm reduction in the management protocol among substance users.

Poly substance use has been identified as a major concern globally due to its sequel of overdose-death crises.^29^ The rate of poly-substance use reported in the index study is alarming, occurring in as high as almost two-thirds of the patients. Similar findings have been documented.^21^,^26^,^27^ The finding of poly substance use being significantly higher in males is expected. It is important to note that poly substance use does not necessarily imply multi-dependence, as ASSIST, the screening tool adopted in this study detects number of substances use but does not establish the severity of use. Also, the UNODC reports similar reported higher substance use among males than females (20% versus 7%).9Nonetheless, the need to institute interventions to curtail such practices even among females should not be overlooked.

There was a significant negative correlation between age and number of substances being abused, which implies that as age increases, the number of substances being used decreases. This is consistent with a review that noted lower substance use as people advance in age.^1^ It is possible that the experience of detrimental effects among older persons may cause them to limit the number of substances being used. Although the index study did not explore the reasons for this observed pattern, we advocate that further studies employing mixed methods should be carried out to fill this gap in knowledge.

Notably, the findings of present study highlight the need for the enforcement of regulation on prescription drugs and review of the drug control laws in Nigeria. Also, the government should impose high excise duty on alcohol and other substances that are commonly used, such as cannabis, opioids and cigarettes to make them less affordable. However, for nations where some substances have been legalized, the need to investigate poly substance use and its effects to guide decision making is hereby advocated. The need for mental health experts to herald the dangers of substance use cannot be overemphasized.

The use of a single centre in our study could limit the external validity and its findings may not reflect the pattern of use in the community. The study did not explore substance quantity, substance duration of use, triggering factors and possible complications among the substance users.

## Conclusion

An overwhelming majority of patients receiving drug rehabilitative service take alcohol. Other commonly used substances include cannabis and prescription opioids. Poly substance use was not uncommon; it was significantly higher among males, and younger population. There is a need to modify alcohol advertisements to include its negative sequel, public sensitization on prevention of substance use, as well as the adoption of the harm reduction approach among substance users. Health warning labels on alcohol adverts and regulation of access to opioids are also advocated.
